# Effects of repeated sprinting on hamstring shear modulus pattern and knee flexor neuromuscular parameters

**DOI:** 10.1038/s41598-023-38861-9

**Published:** 2023-08-03

**Authors:** Ricardo Pimenta, Tomás Lopes, José Pedro Correia, António Prieto Veloso

**Affiliations:** 1 CIPER, Faculdade de Motricidade Humana, Universidade de Lisboa, Estrada da Costa, 1499-002 Cruz Quebrada - Dafundo, Lisboa, Portugal; 2 Research Center of the Polytechnic Institute of Maia (N2i), Maia Polytechnic Institute (IPMAIA), Castêlo da Maia, 4475-690 Maia, Portugal; 3 Department of Biochemistry, King’s College London, Strand, London, UK

**Keywords:** Biophysics, Physiology, Materials science

## Abstract

The purpose of the present study was to examine the acute effects of a maximum repeated sprint protocol on (1) hamstring shear modulus and (2) knee flexor neuromuscular parameters such as peak torque (PT) and rate of torque development (RTD). Muscle shear modulus was assessed in 18 healthy males using shear wave elastography at rest and during 30° isometric knee flexion at 20% of maximal voluntary isometric contraction, before and after a 10 × 30 m repeated sprint protocol. There was a 9% decrease in average speed between the fastest and slowest sprint (p < 0.001; *d* = 2.27). A pre-post decrease was observed in PT (p = 0.004; η^2^_p_ = 0.399) and in the 0–50 ms (p = 0.042; η^2^_p_ = 0.222), and 50–100 ms (p = 0.028; η^2^_p_ = 0.254) RTD periods. For the active shear modulus, the only significant change after the sprint task was in the biceps femoris long head (BFlh) with an increase of 10% (Pre: 26.29 ± 8.89 kPa; Post: 28.93 ± 8.31 kPa; p = 0.015; *d* = 0.31). The present study provides evidence that repeated sprinting leads to significant decreases in average speed, PT, early RTD (0–50 ms; 50–100 ms), and to an increase in BFlh active shear modulus without changing the shear modulus of the other hamstrings muscles.

## Introduction

The hamstrings are a muscle group which acts simultaneously at two joints by participating in knee flexion and hip extension. The hamstrings continue to draw attention in the sports medicine/science field due to the high number of injuries that continues to increase^1^ with fatigue being suggested as a possible risk factor^2^. Muscle fatigue involves progressive mechanical changes during exercise that culminate in a decrease in maximum and rate of force production^3^. This decrease is accompanied by changes in the neuromuscular system in parameters such as peak torque (PT) and the rate of torque development (RTD). PT is an indicator of peripheral fatigue, which is most strongly influenced by morphological features such as the cross-sectional area^4^. RTD is an indicator of supraspinal fatigue which is related to motor unit recruitment, especially in the early phase^5^. Previous studies demonstrated that fatigue has an impact on knee flexors PT after running sprints^6^, on knee flexors and extensors PT without changes of explosive torque after ~ 90 min of the Loughborough Intermittent Shuttle Test^7^, and on RTD after rapid 30 maximal knee extension and flexion repetitions at 180° s^−1^^3^. There is also evidence that fatigue changes the neuromuscular coordination mechanism and causes adaptations of the control strategy over synergistic muscles, i.e., a load sharing strategy^8^. However, few studies have examined how local fatigue affects the load sharing strategy between hamstring muscles^9,10^. It is likely that the control strategy required for the hamstrings also changes with fatigue induction^9,10^.

Previous studies have reported changes in the hamstrings’ load sharing metabolic response using magnetic resonance imaging (i.e., T2 relaxation, which reflects the metabolic activity of the muscle) after knee flexion (KF) dynamic contraction until failure^10,11^. Another method to evaluate the contribution pattern of synergistic muscles is through changes in shear modulus measured by shear wave elastography^12^. Since changes in active shear modulus (i.e., measured during muscle contraction) are correlated with changes in torque production during isometric submaximal contractions^13^, it is possible to use its measurement to determine which muscle provides a greater change in contribution in such tasks, thus detecting the load sharing strategy^12^. Moreover, it has been reported that a decrease in active shear modulus reflects changes in localized muscle fatigue^14,15^ based on a hypothetical decreased ability of the contractile element to generate sarcomere cross-bridges^14,15^. It has been also reported that submaximal and prolonged fatiguing exercises, as well as fatiguing protocols with intermittent contractions and higher contraction intensities, produce a highly variable load sharing pattern^12,14–16^. Regarding the hamstrings, Mendes et al.^9^ reported a decrease in semitendinosus (ST) active shear modulus without corresponding changes in the biceps femoris long head (BFlh) during a fatigue task and suggested that the latter muscle had a greater relative contribution to KF torque production after the onset of fatigue. Moreover, a previous study assessed all the hamstring muscles also using a KF task until exhaustion at the same position, reporting similar results, with a decrease in ST and increase in BFlh contribution without changes in SM and BFsh, reinforcing the idea of this pattern in this position^17^. Together these studies may explain why BFlh has a higher incidence of injury compared to the ST^9,17^. Indeed, it has been suggested that the risk of sustaining a first-time hamstring injury increases significantly if the biceps femoris is activated beyond 10% of its metabolic resting state or if the ST did not participate sufficiently (relative contribution < 34% threshold) during a prone leg curl^11^. However, previous studies^9–11,17^ have evaluated hamstring load using KF tasks; such single-joint tasks have a low functional relevance when compared to an activity such as sprinting^18^, thus it is possible that the load sharing is different in situations where a higher intensity hamstring contraction is needed (such as in sprinting). Measuring muscle shear modulus before and after sprinting (since it is not possible to measure during the task) may thus provide previously undetected changes in the relative load between hamstring muscles with the induction of fatigue. This is especially relevant given the task dependency of fatigue patterns^12,15^. It is also known that the hamstrings play an important role in sprinting^18^, and two of the mechanical determinants of sprint performance are greater hamstring activation just before ground contact and a greater eccentric hamstring torque production, which helps generate greater horizontal force^18^.

The present study primarily aims to (1) determine the effect of a repeated sprint protocol on hamstring shear modulus pattern; (2) determine the effect of a repeated sprint protocol on the neuromuscular parameters (PT and RTD). We hypothesized that (1) the BFlh and ST active shear modulus would increase and decrease, respectively, showing a load sharing strategy; (2) neuromuscular parameters would be affected by the sprint protocol, with decreases in PT and RTD.

## Results

In respect to the shear modulus, the area of the elastogram window and the filling (number of pixels used for shear modulus measurement) was very high (BFlh = 98.1 ± 0.1%; BFsh = 97.5 ± 0.3%; SM = 93.4 ± 2.1%; and ST = 99.5 ± 0.1%), and large elastography map areas were obtained (BFlh = 5.0 ± 0.5 cm^2^, BFsh = 4.7 ± 0.8 cm^2^; SM = 4.7 ± 0.8 cm^2^; and ST = 5.1 ± 0.3 cm^2^).

### Sprint performance

**Figure 1:**
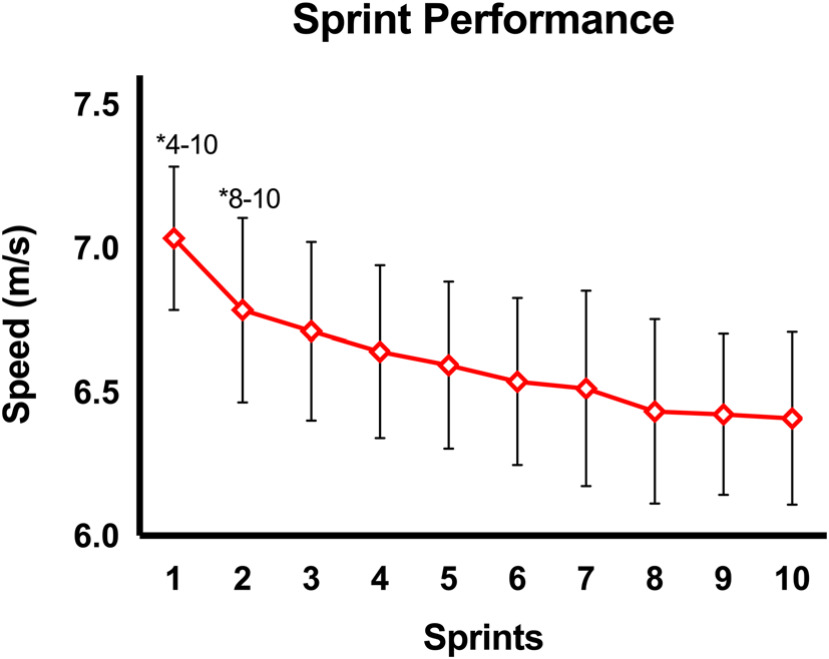
Changes in average speed during the repeated sprinting task. Error bars represent one standard deviation. *Significant difference between the current sprint and each subsequent sprint indicated (p < 0.05).

The fatigue effects on sprint performance are shown in Fig. 1. A significant difference in speed was seen across sprints (p < 0.001; η^2^_p_ = 0.288), with an overall decrease from the fastest and slowest sprint of 9%. The post-hoc tests showed differences between the first and the fourth (p = 0.005; *d* = 0.88), fifth (p < 0.001; *d* = 1.63), sixth (p < 0.001; *d* = 1.85), seventh (p < 0.001; *d* = 1.74), eighth (p < 0.001; *d* = 2.11), ninth (p < 0.001; *d* = 2.29), and tenth sprints (p < 0.001; *d* = 2.27), and between the second and the eighth (p = 0.025; *d* = 1.11), ninth (p = 0.017; *d* = 1.20), and tenth sprint (p = 0.010; *d* = 1.22).

### Neuromuscular parameters

Regarding the analysis of the neuromuscular parameters (Table 1) significant differences were observed in PT between limbs (p = 0.021; η^2^_p_ = 0.275) and pre vs. post (p = 0.004; η^2^_p_ = 0.399), but no interaction effect was present (p = 0.534, η^2^_p_ = 0.023). A significant pre-post difference was seen for RTD 0–50 ms (p = 0.042; η^2^_p_ = 0.222), but not between limbs (p = 0.737; η^2^_p_ = 0.007), and their interaction effect was also not significant (p = 0.452; η^2^_p_ = 0.034). Similarly, RTD 50–100 ms was significantly different between pre vs. post (p = 0.028; η^2^_p_ = 0.254) but not between limbs (p = 0.462; η^2^_p_ = 0.032), and their interaction was not significant (p = 0.224; η^2^_p_ = 0.086). Regarding RTD 150–200 ms, no significant differences were found for the instant factor (p = 0.352; η^2^_p_ = 0.051), limb factor (p = 0.143; η^2^_p_ = 0.122), or their interaction (p = 0.816; η^2^_p_ = 0.003). Finally, no differences were observed in TU-RTDmax between pre vs. post (p = 0.309; η^2^_p_ = 0.061), limbs (p = 0.745; η^2^_p_ = 0.006), or their interaction (p = 0.939; η^2^_p_ < 0.001).

**Table 1:** Neuromuscular parameters values for peak torque, rate of torque development at 0–50 ms (RTD 0–50), 50–100 ms (RTD 50–100), and 150–200 ms (RTD 150–200) and time until maximum RTD (TU-RTDmax) before and after the sprint task.

	Instant		Limb		Instant (pre vs. post)		Limb (left vs right)		Instant × limb interaction	
	Pre	Post	Left	Right	p	η^2^_p_	p	η^2^_p_	p	η^2^_p_
Peak Torque (N⋅m)	117.3 ± 18.9	106.3 ± 18.9	115.3 ± 19.9	108.3 ± 17.0	0.004	0.399	0.021	0.275	0.534	0.023
RTD 0–50 (N⋅m/s)	410.4 ± 149.7	348.6 ± 96.9	376.2 ± 111.0	382.7 ± 125.1	0.042	0.222	0.737	0.007	0.452	0.034
RTD 50–100 (N⋅m/s)	644.0 ± 126.1	573.7 ± 130.5	617.9 ± 113.4	559.9 ± 132.5	0.028	0.254	0.462	0.032	0.224	0.086
RTD 150–200 (N⋅m/s)	327.0 ± 95.9	310.6 ± 51.7	329.5 ± 77.4	308.1 ± 70.8	0.352	0.051	0.143	0.122	0.816	0.003
TU-RTDmax (s)	0.073 ± 0.013	0.078 ± 0.017	0.076 ± 0.017	0.075 ± 0.013	0.309	0.061	0.745	0.006	0.939	< 0.001

Data are presented as mean ± standard deviation.

*PT* peak torque, *RTD 0–50* rate of torque development between 0 and 50 ms, *RTD 50–100* rate of torque development between 50 and 100 ms, *RTD 150–200* rate of torque development between 150 and 200 ms, *TU-RTDmax* time until reaching the maximum value of rate of torque development (s), *p* p value, *η*^*2*^_*p*_ partial eta squared. Data are presented as mean ± standard deviation.

### Active shear modulus

**Figure 2:**
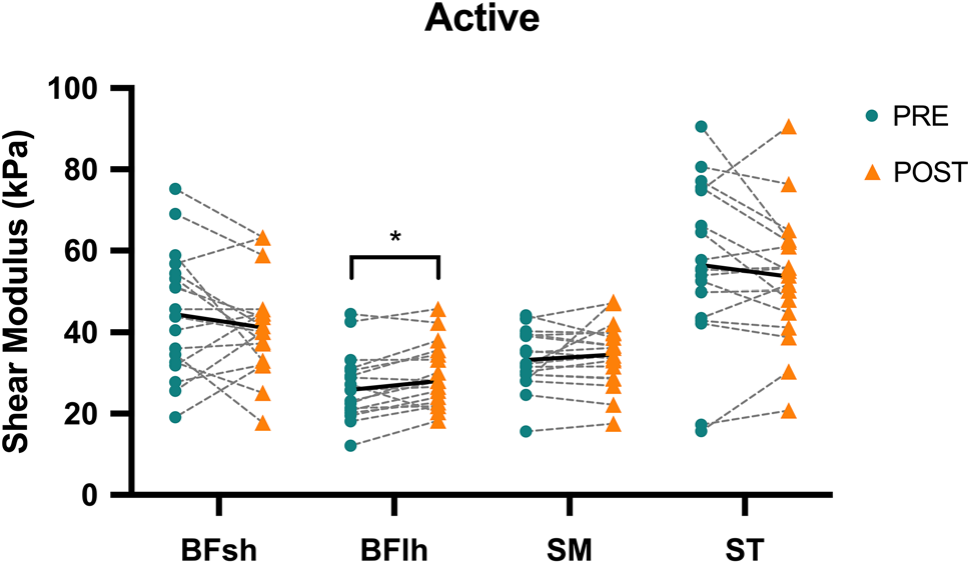
Acute effects of a repeated sprint protocol on the active shear modulus of the biceps femoris long head (BFlh), biceps femoris short head (BFsh), semimembranosus (SM), and semitendinosus (ST). Error bars represent one standard deviation. *Significant difference between pre vs. post (p < 0.05).

Regarding active shear modulus measurements (Fig. 2), differences were identified between muscles (p < 0.001; η^2^_p_ = 0.528), but not between pre vs. post (p = 0.518; η^2^_p_ = 0.028) nor an interaction effect (p = 0.170; η^2^_p_ = 0.111). However, post-hoc analyses showed a significant increase for the active shear modulus of the BFlh between pre vs. post (Pre = 26.29 ± 8.89 kPa; Post = 28.93 ± 8.31 kPa; p = 0.015; *d* = 0.31). Moreover, post-hoc comparisons between muscles demonstrated pre-task differences between the BFlh and BFsh (BFlh = 26.29 ± 8.89 kPa; BFsh = 44.50 ± 16.59 kPa; p = 0.005; *d* = 1.37), BFlh and ST (BFlh = 26.29 ± 8.89 kPa; ST = 55.94 ± 21.65 kPa; p < 0.001; *d* = 1.79), and SM and ST (SM = 33.94 ± 7.80 kPa; ST = 55.94 ± 21.65 kPa; p = 0.005; *d* = 1.36). Post-task, significant differences were seen between the BFlh and ST (BFlh = 28.93 ± 8.31 kPa; ST = 53.0 ± 17.78 kPa; p < 0.001; *d* = 1.73), BFsh and ST (BFsh = 41.31 ± 13.07 kPa; ST = 53.0 ± 17.78 kPa; p = 0.029; *d* = 0.75) and SM and ST (SM = 34.18 ± 7.86 kPa; ST = 53.0 ± 17.78 kPa; p = 0.008; *d* = 1.37). The standard error of measurement (SEM) was calculated for each muscle (BFlh = 3.8 kPa; BFsh = 5.4 kPa; SM = 3.1 kPa; ST = 6.6 kPa). An inter-individual analysis for each muscle (Table 2) was conducted to reflect the post–pre changes beyond the SEM.

**Table 2:**
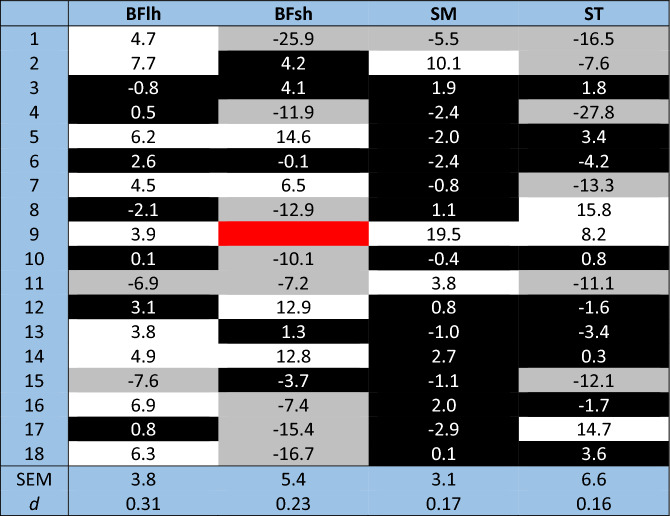
Post–pre differences in active shear modulus (kPa) for a repeated sprint task across all hamstring muscles.

White cells correspond to an increase while the gray cells correspond to a decrease after the task, black cells change within the standard error of measurement and red cells correspond to a missing value.

*BFlh* biceps femoris long head, *BFsh* biceps femoris short head, *SM* semimembranosus, *ST* semitendinosus, *SEM* standard error of measurement, *d* Cohen’s *d* effect size.

## Discussion

To the best of our knowledge, this is the first study to examine the acute effects of fatigue before and after repeated sprinting on the hamstring load sharing pattern as measured by the active shear modulus. The main findings were: (1) BFlh shear modulus increased after the fatigue task without changes in the other muscles; (2) PT and early-phase RTD values decreased after the sprint fatigue task.

As hypothesized, a decrement in the neuromuscular parameters was observed after the sprint task, impacting PT and early-phase RTD. Importantly, early RTD is largely dependent on motor unit recruitment speed and maximal discharge rate and is related to supraspinal fatigue^5,19^. On the other hand, late RTD is strongly correlated with PT and therefore seems to depend more on structural factors, being an indicator of peripheral fatigue which is influenced by morphological features such as the cross-sectional area^4,20,21^. On average, early RTD decreased more than late RTD, suggesting that early contraction phases may be particularly sensitive to neuromuscular fatigue induced by repeated sprinting^22^. Thus, the results of the present study indicate that a repeated sprint task affects the biomechanical system probably through changes both at the peripheral (muscle) and supraspinal levels.

Some studies have examined the quadriceps and hamstrings load sharing pattern^9,12,14–16,23,24^. Concerning the hamstrings, Mendes et al. noted that the ST generally displayed a greater active shear modulus compared to BFlh, especially at low intensities (i.e. < 40% MVIC)^23^, and that the active shear modulus of the ST decreases without a corresponding change in the BFlh, thus increasing the BFlh/ST ratio^9^ after a KF fatigue task at 20% of MVIC^9^. Together, these two studies indicate that BFlh/ST active shear modulus ratio is sensitive to both contraction intensity and fatigue levels. In the present study, the load sharing active shear modulus hypothesis was not confirmed since only the BFlh showed a significant increase (10%), with the decrease of BFsh (− 7.3%), ST (− 5.0%) and the increase of SM (3.9%) not reaching statistical significance. It should also be noted that the BFlh showed a significant difference that is within the SEM, which could indicate a statistical but not physiological relevance. However, the inter-individual sharing response analysis (the absolute post–pre differences) on the hamstrings muscles as shown in Table 2, detected a different behavior between individuals. Indeed, a tendency for a shear modulus increase was seen in the BFlh in 9/18 individuals, while 8/17 and 6/18 individuals showed decreased shear modulus of the BFsh and ST, respectively. These findings are partially in accordance with previous literature, as Chen et al. (2018) reported a higher shear wave velocity for the BF muscle after a sprint task^25^, although since the BFlh was the only measured muscle and was measured in a rest condition it is not possible to compare the contribution across the other hamstrings muscles and with an active measurement. Furthermore, Timmins et al.^26^ reported that the medial hamstrings (SM and ST) concentric and eccentric myoelectrical activity was unaffected, while a significant decrease in eccentric (10%) but not concentric BFlh activity was seen. It should be noted, however, that Timmins et al.^26^ used a different sprint protocol (three sets of six 30-m sprints (10 m for acceleration + 20 m) with rest periods ranging between 90 and 240 s) as well as a different testing position and measurement method (isokinetic evaluation); sprint speed differences were not reported. In our study, we found a similar decrease of 10% on isometric knee flexion PT and a 9% decrease in sprint speed. The decreased BFlh myoelectric activity that was only seen on eccentric and not concentric contractions in Timmins et al.’s study could also be due to differences in the sprint and measurement protocols, since they used isokinetic eccentric contractions after the fatigue task, which are known to lead to a lesser degree of muscle activity. Indeed, as reported by the same authors, the BF experiences larger peak active strains during the terminal swing phase of running than the medial hamstrings (SM and ST)^27^, and these may predispose the former to greater muscle damage^28^.

Additionally, exercise-induced muscle damage has been shown to result in significant reductions in voluntary activation^29,30^ and EMG^31^, which may increase afferent feedback and lead to a decreased myoelectrical activity of the BF in an attempt to minimize exposure to the damaging stimulus^32^. It is interesting to note that the BFlh was the only muscle affected by repeated sprinting, suggesting that both methodologies [SWE and electromyography (EMG)] are sensitive to these changes. Repeated sprinting therefore leads not only to a change in this recruitment pattern in eccentric contractions (the primary action of the hamstrings in high-velocity sprints) but also to a greater active shear modulus after the task. It should be noted that our measurements were taken in a different type of contraction (isometric); however, higher shear modulus values are expected in an eccentric action, which would amplify this result. The active shear modulus has also been reported to increase after eccentric damaging exercise^33,34^. Considering that the BFlh is the muscle most commonly affected by sprint-related injuries and that sprinting seems to place a greater load on the BFlh^35^, increasing injury risk to this muscle, the relevance of these changes need to be analyzed in a more detailed manner (e.g., combined with other methodologies such as EMG during sprints to compare data with SWE) in order to clarify their role in injury risk. Despite these results, we cannot conclude that fatigue induced by repeated sprinting affects the hamstrings’ load sharing mechanism as evaluated by the shear modulus, since an increase was only detected in the BFlh. However, it could be interesting to analyze participants with and without previous hamstring injuries to confirm whether a greater BFlh active shear modulus is seen after repeated sprinting on injured participants. This warrants further investigation.

In relation to the analyses between muscles, a lower BFlh and SM shear modulus was seen in comparison to BFsh and ST, which can be due to our test position which primarily mobilized the knee (identical to a prone leg curl), since a greater recruitment of ST and BFsh seems to occur in knee-dominant exercises (e.g., prone leg curl)^10,36,37^. Furthermore, it should be noted that the pre-task significant differences between muscles shear modulus were not found post-task. Indeed, despite the non-significant pre vs. post difference, the largest decrease was seen in the BFsh causing its significant pre-task difference relative to the BFlh to become non-significant post-task. Additionally, there was both a post-task decrease in BFsh shear modulus and an increase in BFlh, which led to a significant post-task difference between the BFsh and ST. It is unknown whether these differences within the BF (decrease in BFsh and increase in BFlh) have physiological relevance for an increased injury risk on BFlh muscle; the relevance of these changes need to be analyzed in greater detail.

This study has some limitations. Firstly, since the study sample only included young males which performed resistance training, the results cannot be generalized to other groups. Secondly, shear modulus measurements were only performed in one leg. It should be noted, however, that a symmetrical inter-limb hamstring shear modulus pattern has been reported in the same position using an isometric step contraction with multiple intensities^23^. Thirdly, the measurements were performed at a single site within each muscle, and it is known that active shear modulus differences exist within the muscle^38^. Thus, we are unaware whether the fatigue effects on muscle shear modulus are also region-dependent. Fourthly, it is necessary to consider the contribution of other muscles such as the gastrocnemius, sartorius, and gracilis to the sprint task but also to knee flexion MVIC and shear modulus assessment. Fifthly, we assumed that the load sharing measured in the present study was a direct result of the repeated sprinting task; however, the measurements were performed in knee flexion, which involves different demands and recruitment patterns.

## Conclusion

The present study provides evidence that repeated sprinting impacts the average sprint speed and neuromuscular parameters such as PT and early RTD (0–100 ms). Furthermore, a significant active shear modulus increase was only seen for the BFlh. Nevertheless, more research is still needed to properly understand the association between sprint-induced fatigue and the hamstrings’ load sharing pattern, especially during sprinting. Therefore, future research should consider analyzing the load sharing strategy as measured by changes in shear modulus during sprinting; further developments in SWE technology will be needed in this regard. It would also be worthwhile to compare these measurements between injured and uninjured participants to verify whether load sharing patterns differ.

## Methods

### Participants

Eighteen resistance-trained male adults who perform strength and conditioning training at least four times per week (age: 25.2 ± 4.8 years; height: 177.6 ± 6.4 cm; body mass: 79.1 ± 10.8 kg) without a history of hamstring strain injury in the last 2 years were invited to participate in this study. The sample size was estimated using G*Power software (Heinrich-Heine Universität, Dusseldorf, Germany) for a repeated-measures within-subject ANOVA, with data from a previous study using an effect size of 0.46 and a statistical power of 95%, resulting in a sample size of 18 participants^17^. For convenience, only males were recruited because they often present less thigh subcutaneous and intramuscular adipose tissue than women, which allowed for a better shear modulus evaluation. Participants were instructed to avoid any strenuous activities 24 h before the test to minimize confounding factors. All participants read and signed an informed consent prior to participating in the study. The Ethical Committee at the Faculty of Human Kinetics at the University of Lisbon approved the study (#5/2021). All methods were performed in accordance with the Declaration of Helsinki.

### Equipment and variables

#### Shear wave elastography

Hamstrings shear modulus was assessed using two similar ultrasound scanners (Aixplorer, v11; Supersonic Imagine, Aix-en-Provence, France; Aixplorer, v12; Supersonic Imagine, Aix-en-Provence, France) in SWE mode [musculoskeletal preset, penetrate mode, smoothing level 5, opacity 100%, scale: 0–800 kPa for active (i.e. during contraction) condition, coupled with a linear transducer array (SL10-2, 2–10 MHz. Super Linear, Vermon, Tours, France). The push frequency that generated the elastogram window was set automatically by the ultrasound equipment to approximately 1 Hz (range 0.8–1.4 Hz)^39^. It should be noted that the device output is Young’s modulus, which corresponds to a linear displacement, whereas the shear modulus corresponds to an angular displacement. Therefore, to estimate the shear modulus, it is necessary to have some considerations. First, the ultrasound scanner measures shear wave speed using the equation^39^:

$$E = {3}\rho V_{s}^{2}$$

where *E* the Young’s modulus, *ρ* is the muscle mass density (1000 kg/m^3^) and $V_{s}^{2}$ is the shear wave velocity. The ultrasound scanner also provides the corresponding Young's modulus which is related to shear modulus (*G*), since *G* = *ρ *$V_{s}^{2}$; therefore, shear modulus can be estimated using the following equation^39^:

$$E\approx 3G$$

**Figure 3:**
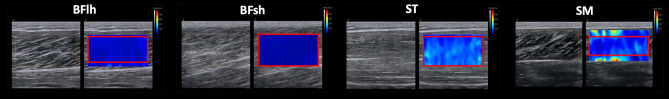
Shear modulus assessment at 20% of MVIC before the fatigue task for biceps femoris long head (BFlh), biceps femoris short head (BFsh) semimembranosus (SM), semitendinosus (ST). The red line within the elastogram window corresponds to the area used for calculating the average shear modulus.

Since muscle strains occur mainly due to a shear force on the muscle fibers^40^, shear modulus is often used in muscle rigidity analysis to evaluate injured muscle tissue. Shear modulus calculations assume isotropy of the underlying tissue; it is known, however, that muscle is anisotropic, since the mechanical properties of muscle fibers are direction-dependent^41^. The anisotropy of skeletal muscle therefore requires a transducer orientation in SWE aligned with the fascicles orientation (since the shear waves preferably propagate in their direction), to obtain accurate shear modulus values^42^. Furthermore, from a physiological standpoint, the shear wave speed and associated shear modulus are perhaps most relevant when aligned with muscle fibers, as this may represent the mechanism by which mechanical forces are transmitted through tissue, allowing skeletal muscles to perform work^43^. The muscles’ region of interest where the muscle shear modulus was assessed corresponded to the largest cross-sectional area where the hyperechoic lines delineating the muscle fascicles (i.e. perimysial membranes) were well visualized (Fig. 3). Therefore, measurements were obtained with transducers placed at ~ 43% (SM), ~ 55% (BFlh), ~ 55% (ST), and ~ 20% (BFsh) of the distal-to-proximal femur length^23^. Care was taken to align the transducer with the muscles fascicles’ orientation and to perform minimal pressure during the measurements. To ensure the same ROI was measured before and after the repeated sprint task, plastic casts were fixed to the skin with double-sided tape and maintained during the task. To verify data quality, elastography map areas were measured.

#### Dynamometry

The knee flexor torque was measured at a sampling rate of 1000 Hz using custom-made equipment^9,44^. Participants were placed in the prone position, with the hips in neutral anatomical position, knees flexed at 30° (0° = full extension) and the ankle in 15° of plantar flexion, as previously reported. This position allows for the assessment of muscle shear modulus with minimal passive tension^45^. Both feet were fixed in individual foot holders each containing a force transducer (Model STC, Vishay Precision, Malvern, PA) at the heel level to collect the linear force perpendicular to the leg orientation and with the ankle at 90°. Force data were amplified (Model UA73.202, Sensor Techniques, Cowbridge, UK), digitally converted (USB-230 Series, Measurement Computing Corporation Norton, MA), recorded using the DAQami software (v4.1, Measurement Computing Corporation, Norton, MA), and multiplied by the perpendicular distance between the force transducer center and the femoral lateral condyle in order to estimate the knee torque. Visual feedback of force production was provided to individuals during the assessments. Moreover, ​​participants were instructed to exert force “as fast and strong as possible”, to obtain both PT and RTD^46^. The RTD was estimated for the following time intervals considering force onset: 0–50 ms, 50–100 ms, 150–200 ms, and time until maximum RTD (TU-RTDmax). The parameter TU-RTDmax was used to detect if fatigue also changes the time to reach the maximum RTD since a previous study indicated a greater magnitude of responses of this parameter to a fatigue state compared with MVIC^22^.

#### Sprint performance

Sprint performance was evaluated by a maximal repeated sprint protocol, with participants departing from a two-point stance and positioned 1 m behind the photocells. The average sprint speed was recorded using four photocells placed at the start and finish and data was processed using the Chronojump software (version: 2.1.1–16, Chronojump Boscosystem, Barcelona, Spain).

### Protocol

Participants performed an indoor repeated sprint protocol, so wind had no effect on performance. To ensure no changes in force production occurred, the examiner and participants had visual force production feedback. Then, individuals were asked to perform 10 submaximal knee flexion contractions at a self-perceived low intensity with a visual guide at 20% MVIC to prepare and familiarize with the equipment for the maximum voluntary isometric contraction (MVIC) evaluation, which consisted of two 3-s trials with 30 s of recovery between trials. Participants were instructed to perform the trials “as fast and hard as possible”^46^. Based on the highest PT on the tested limb, individuals familiarized themselves with the 20% of MVIC through trials using visual feedback. Subsequently, the active shear modulus was then measured twice for each muscle at 20% of MVIC. Each trial lasted ~ 30 s. The intensity of 20%MVIC was chosen based on a previous study^17^ since participants demonstrated a greater difficulty to maintain the minimal contraction time for a valid measure of 20 s at 40% MVIC after a monoarticular fatigue task; since sprinting involves higher demands, a conservative approach was used and the 20% intensity was chosen. Moreover, a good reliability has been observed at this intensity^23^ and it is possible to compare the results with previous literature^9,17,23^.

After the pre-task active shear modulus measures, a standardized warm-up protocol for sprinting was performed, composed by 5 min of running on a treadmill at 2 m/s and 3 × 30 s sets of low, medium, and high skipping. Immediately after the warm-up, a 10 × 30 m repeated sprint task was performed. Then, post-task active shear modulus measurements were conducted followed by two MVIC trials. Both the order of muscle measurement and the tested limb (9 measurements on the left and 9 on the right for the shear modulus analysis; all participants were right-limb dominant) were randomized.

### Data processing

Shear wave elastography data were processed using automated Matlab routines (The Mathworks Inc., Natick, MA)^9^. For the shear modulus calculation, each clip exported from Aixplorer’s software was sequenced in .jpeg images. Image processing converted each pixel of the color map into a value of the Young’s modulus based on the recorded color scale. The largest ROI in the elastogram window was determined by avoiding aponeuroses and tissue artifacts (e.g. vessels) and the values were averaged to obtain a representative muscle value. Within each trial, the most stable Young’s modulus values over ~ 20 s in the active condition were averaged and divided by 3 to better represent the muscle shear elastic modulus^39^. The shear modulus of each muscle was considered for analysis.

Neuromuscular parameters such as PT, 0–50 ms, 50–100 ms, 150–200 ms RTD, and TU-RTDmax were determined using automated Matlab routines (The Mathworks Inc., Natick, MA). The onset of force production was defined by visual detection and using a threshold-based mathematical algorithm^47,48^. In brief, after selecting the onset by visual detection, the threshold-based model verified this within specific time frame conditions, identifying the point where the torque value reached three standard deviations above the baseline value. It should be noted that the visual detection merely supports the detection of the onset by the mathematical model, serving as an indication of which approximate time frame to search for the onset, with the model then searching around (before and after) the visually-detected onset for a more accurate onset. In case a more accurate onset would be detected by the algorithm in relation to the visual detection, this would then be selected as the final onset.

### Statistical analysis

Data are presented as mean ± standard deviation. Data analysis was performed using IBM SPSS Statistics 27.0 (IBM Corporation, Armonk, NY). Normality of data distribution was analyzed using the Shapiro–Wilk test.

The effect of the sprint task (10 sprints) was examined by conducting a one-way repeated measures ANOVA [sprint (1–10)] for the average sprint speed variable. The sprint task and limb (right, left) effects, as well as their interaction, were examined by conducting a two-way repeated measures ANOVA [pre vs. post × limb (right, left)] for each neuromuscular parameter. The sprint task and their muscle (BFsh, BFlh, SM and ST) effects, as well as their interaction, were examined by conducting a two-way repeated measures ANOVA [pre vs. post x muscle (BFsh, BFlh, SM, and ST)] on the active shear modulus. Post-hoc analysis was conducted using Bonferroni correction to determine the differences within each factor.

Cohen’s *d* effect sizes were determined and classified as small (*d* = 0.2–0.5), medium (0.5 ≥ *d* < 0.8), and large (*d* ≥ 0.8) for normally distributed data based on benchmarks suggested by Cohen ^49^. The partial eta square (η^2^_p_) values were reported as a measure of the effect size of the ANOVA’s findings, classified as small (η^2^_p_ = 0.01–0.05), medium (η^2^_p_ = 0.06–0.013), and large (η^2^_p_ > 0.14) effects^49^. The SEM was calculated and determined as a methodological error outcome. Statistical significance was set at p < 0.05.

## Data Availability

The datasets generated during and/or analyzed during the current study are available from the corresponding author on reasonable request.

## References

[1] Ekstrand J (2022). Hamstring injury rates have increased during recent seasons and now constitute 24% of all injuries in men’s professional football: The UEFA Elite Club Injury Study from 2001/02 to 2021/22. Br. J. Sports Med..

[2] Woods C (2004). The Football Association Medical Research Programme: An audit of injuries in professional football—analysis of hamstring injuries. Br. J. Sports Med..

[3] Zhang Q, Morel B, Trama R, Hautier CA (2021). Influence of fatigue on the rapid hamstring/quadriceps force capacity in soccer players. Front. Physiol..

[4] Carroll TJ, Taylor JL, Gandevia SC (2017). Recovery of central and peripheral neuromuscular fatigue after exercise. J. Appl. Physiol..

[5] Johnson ST, Kipp K, Norcross MF, Hoffman MA (2015). Spinal and supraspinal motor control predictors of rate of torque development. Scand. J. Med. Sci. Sports.

[6] Baumert P (2021). Neuromuscular fatigue and recovery after strenuous exercise depends on skeletal muscle size and stem cell characteristics. Sci. Rep..

[7] Behan FP, Willis S, Pain MTG, Folland JP (2018). Effects of football simulated fatigue on neuromuscular function and whole-body response to disturbances in balance. Scand. J. Med. Sci. Sports.

[8] Stutzig N, Siebert T (2015). Muscle force compensation among synergistic muscles after fatigue of a single muscle. Hum. Mov. Sci..

[9] Mendes B (2020). Effects of knee flexor submaximal isometric contraction until exhaustion on semitendinosus and biceps femoris long head shear modulus in healthy individuals. Sci. Rep..

[10] Schuermans J, Van Tiggelen D, Danneels L, Witvrouw E (2014). Biceps femoris and semitendinosus—teammates or competitors? New insights into hamstring injury mechanisms in male football players: A muscle functional MRI study. Br. J. Sports Med..

[11] Schuermans J, Van Tiggelen D, Danneels L, Witvrouw E (2016). Susceptibility to hamstring injuries in soccer: A prospective study using muscle functional magnetic resonance imaging. Am. J. Sports Med..

[12] Bouillard K, Hug F, Guével A, Nordez A (2012). Shear elastic modulus can be used to estimate an index of individual muscle force during a submaximal isometric fatiguing contraction. J. Appl. Physiol..

[13] Hug F, Tucker K, Gennisson J-L, Tanter M, Nordez A (2015). Elastography for muscle biomechanics: Toward the estimation of individual muscle force. Exerc. Sport Sci. Rev..

[14] Bouillard K, Jubeau M, Nordez A, Hug F (2014). Effect of vastus lateralis fatigue on load sharing between quadriceps femoris muscles during isometric knee extensions. J. Neurophysiol..

[15] Morel B (2019). Reduced active muscle stiffness after intermittent submaximal isometric contractions. Med. Sci. Sports Exerc..

[16] Chalchat E (2020). Changes in the viscoelastic properties of the vastus lateralis muscle with fatigue. Front. Physiol..

[17] Pimenta R (2023). Effects of fatigue on hamstrings and gluteus maximus shear modulus in hip extension and knee flexion submaximal contraction task. Sports Biomech..

[18] Morin J-B (2015). Sprint acceleration mechanics: The major role of hamstrings in horizontal force production. Front. Physiol..

[19] Vecchio AD (2019). You are as fast as your motor neurons: Speed of recruitment and maximal discharge of motor neurons determine the maximal rate of force development in humans. J. Physiol..

[20] Andersen LL, Andersen JL, Zebis MK, Aagaard P (2010). Early and late rate of force development: Differential adaptive responses to resistance training?. Scand. J. Med. Sci. Sports.

[21] Folland JP, Buckthorpe MW, Hannah R (2014). Human capacity for explosive force production: Neural and contractile determinants. Scand. J. Med. Sci. Sports.

[22] D’Emanuele S (2021). Rate of force development as an indicator of neuromuscular fatigue: A scoping review. Front. Hum. Neurosci..

[23] Mendes B (2018). Hamstring stiffness pattern during contraction in healthy individuals: Analysis by ultrasound-based shear wave elastography. Eur. J. Appl. Physiol..

[24] Evangelidis PE (2021). Hamstrings load bearing in different contraction types and intensities: A shear-wave and B-mode ultrasonographic study. PLoS One.

[25] Chen C-H, Ye X, Wang Y-T, Chen Y-S, Tseng W-C (2018). Differential Effects of Different Warm-up Protocols on Repeated Sprints-Induced Muscle Damage. J. Strength Cond. Res.

[26] Timmins RG (2014). Reduced biceps femoris myoelectrical activity influences eccentric knee flexor weakness after repeat sprint running. Scand. J. Med. Sci. Sports.

[27] Thelen DG (2005). Hamstring muscle kinematics during treadmill sprinting. Med. Sci. Sports Exerc..

[28] Garrett WE, Safran MR, Seaber AV, Glisson RR, Ribbeck BM (1987). Biomechanical comparison of stimulated and nonstimulated skeletal muscle pulled to failure. Am. J. Sports Med..

[29] Endoh T, Nakajima T, Sakamoto M, Komiyama T (2005). Effects of muscle damage induced by eccentric exercise on muscle fatigue. Med. Sci. Sports Exerc..

[30] Skurvydas A, Brazaitis M, Kamandulis S, Sipaviciene S (2010). Peripheral and central fatigue after muscle-damaging exercise is muscle length dependent and inversely related. J. Electromyogr. Kinesiol..

[31] Beck TW, Kasishke PR, Stock MS, DeFreitas JM (2012). Neural contributions to concentric vs. eccentric exercise-induced strength loss. J. Strength Cond. Res..

[32] Marqueste T (2004). Eccentric exercise alters muscle sensory motor control through the release of inflammatory mediators. Brain Res..

[33] Heales LJ (2018). Shear-wave velocity of the patellar tendon and quadriceps muscle is increased immediately after maximal eccentric exercise. Eur. J. Appl. Physiol..

[34] Goreau V (2022). Hamstring muscle activation strategies during eccentric contractions are related to the distribution of muscle damage. Scand. J. Med. Sci. Sports.

[35] Askling CM, Tengvar M, Saartok T, Thorstensson A (2007). Acute first-time hamstring strains during high-speed running. Am. J. Sports Med..

[36] Fernandez-Gonzalo R (2016). Individual muscle use in hamstring exercises by soccer players assessed using functional MRI. Int. J. Sports Med..

[37] Bourne MN, Opar DA, Williams MD, Al Najjar A, Shield AJ (2016). Muscle activation patterns in the Nordic hamstring exercise: Impact of prior strain injury. Scand. J. Med. Sci. Sports.

[38] Vaz JR, Neto T, Correia JP, Infante J, Freitas SR (2021). Regional differences in biceps femoris long head stiffness during isometric knee flexion. J. Funct. Morphol. Kinesiol..

[39] Bercoff J, Tanter M, Fink M (2004). Supersonic shear imaging: A new technique for soft tissue elasticity mapping. IEEE Trans. Ultrason. Ferroelectr. Freq. Control.

[40] Kääriäinen M (2000). Integrin and dystrophin associated adhesion protein complexes during regeneration of shearing-type muscle injury. Neuromuscul. Disord..

[41] Brandenburg JE (2014). Ultrasound elastography: The new frontier in direct measurement of muscle stiffness. Arch. Phys. Med. Rehabil..

[42] Gennisson J-L (2010). Viscoelastic and anisotropic mechanical properties of in vivo muscle tissue assessed by supersonic shear imaging. Ultrasound Med. Biol..

[43] Sharafi B, Blemker SS (2010). A micromechanical model of skeletal muscle to explore the effects of fiber and fascicle geometry. J. Biomech..

[44] Pimenta R, Lopes T, Bruno P, Veloso A (2023). Effects of repeated sprints on hamstring active shear modulus pattern and neuromuscular parameters in football players with and without hamstring strain injury history—a retrospective study. Appl. Sci..

[45] Silder A, Whittington B, Heiderscheit B, Thelen DG (2007). Identification of passive elastic joint moment-angle relationships in the lower extremity. J. Biomech..

[46] Maffiuletti NA (2016). Rate of force development: Physiological and methodological considerations. Eur. J. Appl. Physiol..

[47] Li X, Zhou P, Aruin AS (2007). Teager–Kaiser energy operation of surface EMG improves muscle activity onset detection. Ann. Biomed. Eng..

[48] Solnik S, Rider P, Steinweg K, DeVita P, Hortobágyi T (2010). Teager-Kaiser energy operator signal conditioning improves EMG onset detection. Eur. J. Appl. Physiol..

[49] Cohen J (1988). Statistical Power Analysis for the Behavioral Sciences.

